# Tears of Joy as an Emotional Expression of the Meaning of Life

**DOI:** 10.3389/fpsyg.2022.792580

**Published:** 2022-03-08

**Authors:** Bernardo Paoli, Rachele Giubilei, Eugenio De Gregorio

**Affiliations:** ^1^Accademia Delle Tecniche Psicologiche, Turin, Italy; ^2^Istituto per lo Studio Delle Psicoterapie, Rome, Italy; ^3^Link Campus University, Rome, Italy

**Keywords:** dimorphous emotional expressions, tears of joy, meaning of life, semi-structured interviews, narrative analysis

## Abstract

This article describes a research project in which a qualitative research was carried out consisting of 24 semi-structured interviews and a subsequent data analysis using the MAXQDA software in order to investigate a particular dimorphic emotional expression: tears of joy (TOJ). The working hypothesis is that TOJ are not only an atypical expression due to a “super joy,” or that they are only an attempt by the organism to self-regulate the excess of joyful emotion through the expression of the opposite emotion (sadness), but that it is an emotional experience in its own right—not entirely overlapping with joy—with a specific adaptive function. Through the interviews, conducted in a cross-cultural context (mainly in India and Japan), we explored the following possibility: what if the adaptive function of crying for joy were to signal, to those experiencing it, the meaning of their life; the most important direction given to their existence? The material collected provided positive support for this interpretation.

## Introduction

The emotions we experience and express have an adaptive function. They allow us to live better in the surrounding environment and respond accordingly to specific circumstances (e.g., fleeing from danger in response to feeling fear). They are the result of a process, of a largely unconscious process of self-regulation, which modulates their nature, intensity and manifestations. Responses to emotions are almost never as violent and powerful as they could be; as such, it happens rarely that a person “loses their mind” completely ignoring internal and external feedback signals. Emotions are also involved in automatic regulation (Matarazzo and Zammuner, 2009). We make conscious attempts to modify our emotions in order to align them with our goals and interests by asking ourselves whether it is useful to feel fear when facing a certain difficulty. Thus, we can cope with fear by changing the perspective we use to observe the situation. Emotional regulations mean that one is able to modulate their form or mitigate their urgency. More generally, it means that one is able to assess demands from the environment and one’s own resources, and to respond to them in a flexible and adaptive way. Emotions, properly modulated and regulated, improve social interaction and individual wellbeing. Crying is an emotional expression that has both a self-regulatory goal (decreasing tension and recovering psychological balance) and the purpose of attracting the attention of others and requesting their help and support. Crying can also be the emotional expression of the “opposite” emotion: it can occur in situations in which a person experiences great joy. The dimorphous expression of tears of joy (TOJ) is a unique, non-mixed experience: the person experiences only joy, and not joy mixed with sadness. TOJ occurs in very significant situations in people’s lives, especially in correspondence with the perception of a deep connection with others and with episodes of personal success.

These are the findings from the literature, and the starting point for our qualitative research. This research, which started in an unusual way—due to a journey in India and in Japan taken by one of the authors—sought to explore the cultural influence on emotions and emotional expression, and, above all, on the possible links between TOJ and people’s definition of the meaning of life (MOL). Regarding the first point, we know from the literature that there are cultural differences that regulate emotional expression. In collectivist cultures (which exalt “the good of the group before the good of the individual”) social rules seem to lead people to avoid the direct expression of emotions, especially negative ones, which can undermine group harmony; on the other hand, the rules of individualistic cultures (which exalt “the good of the individual before the good of the group”) seem to be more tolerant of the expression of strong emotions (e.g., anger), because of values such as self-affirmation. Interestingly, the emotional expression of crying tends to occur more frequently in democratic countries with individualistic cultures, where there is greater wealth and political freedom. As for the second point—the pivot of the research—the literature says that people who experience a higher level of meaningfulness in their lives are more likely to experience positive moods and greater wellbeing. So, what if the adaptive function of TOJ were to signal the MOL to those experiencing it? In the literature, these two themes have never been related to each other. The present study aims to investigate a possible connection.

### The Adaptive Function of Emotions

From an evolutionary perspective, emotions help humans cope with and adapt to different situations in life (cf. Lazarus, 1991; Plutchik, 2001). When triggered, emotions simultaneously activate different systems—such as perceptual, attentional, and neurophysiological systems—that predispose people to certain behaviors (Tooby and Cosmides, 2008). For example, anger stimulates the activation of the sympathetic nervous system and triggers a flow of energy adequate to support a dynamic response to the situation, which may be self-defense or decompression; fear, on the other hand, induces a state of neurophysiological activation that allows the individual to respond to the initial stimulus through attack, avoidance, or escape. In the literature (Fredrickson, 1998; Ruini, 2017) it has been found that “positive” emotions play important adaptive functions, on par with “negative” ones. Specifically, emotions such as joy, interest, or satisfaction have the ability to modify styles of thought and action, and may expand and improve people’s cognitive, behavioral, and social skills, which will in turn become lasting resources for the future. Fredrickson (1998) further argues that the important adaptive functions of positive emotions lie in their ability to counteract negative emotions and their consequences, leading to improved mental and physical health and motivation to work for one’s wellbeing.

### The Regulatory Function of Emotions: Emotional Self-Regulation

It has been widely attested in the literature that an individual’s ability to self-regulate their emotions is a key feature in maintaining social functioning, physical wellbeing, and mental health (cf. Gross and John, 2003). Emotional regulation can be defined as the ability to cope with, monitor, and modulate one’s own emotional experiences, and refers to behavioral and cognitive processes that coordinate the intensity, duration, and expression of emotions in response to internal and external stimuli (Gross, 1998; Gross, 2007). For example, when we find ourselves in circumstances that trigger fear, we can divert our attention away from those elements of the situation that most disturb us and focus instead on other aspects we perceive as less dangerous in order to regulate the intensity of the emotion felt in that moment. Gross (2001) distinguishes between antecedent-focused and response-focused strategies: antecedent-focused strategies refer to what individuals do before the emotional response tendencies are fully activated (e.g., choosing not to frequent places or people perceived as disturbing in order to avoid the onset of unpleasant emotions); response-focused strategies refer to what people do when the emotional response is in progress (e.g., striving to appear calm rather than angry, thus suppressing the expression of the emotion felt). The different strategies can down-regulate or up-regulate emotional experiences: while down-regulation strategies tend to attenuate the emotional response, up-regulation strategies contribute to enhancing and prolonging it, whether it be a positive emotion triggered by a pleasant event, or a negative emotion generated by an unpleasant event.

Many works in the literature have focused on the regulation of negative emotions, while few studies have focused on understanding how people regulate positive emotions, such as happiness or pride. Positive emotion regulation refers to how humans modulate responses to stimuli in order to enhance and maintain the positive emotional experiences experienced at a given time (Bryant and Veroff, 2007). Some studies (cf. Langston, 1994) show that, when experiencing very positive situations (e.g., victory in a competition; achievement of an important goal) people can implement behaviors designed to protect their state of happiness: for example, sharing good news with friends or relatives is a way to prolong the positive experience (capitalization). Positive emotional regulation strategies include savoring (Bryant, 1989), which is the ability of humans to enhance and intensify positive experiences in their lives (Bryant and Veroff, 2007). Bryant et al. (2005) theorizes three types of savoring: savoring in relation to an impending positive situation (e.g., anticipatory excitement before an enjoyable event); savoring that prolongs and reinforces the positive event experienced in the present moment (e.g., sharing the experience with people important to us); and savoring intense memories of positive experiences in order to relive the emotions associated with them (e.g., remembering the best moments of an event). Bryant and Veroff (2007), identified several strategies that people use to enhance their savoring ability and that are expressed at cognitive, behavioral, and interpersonal levels. These include: memory building, which consists in creating a lasting memory of an event (for example, taking a mental picture of an event, or taking a diary); self-congratulation every time we manage to achieve something important in our lives in order to amplify positive feelings; sharing positive emotion with others; comparing the experience lived with a worse one, to appreciate it more; attentional absorption, which means to stay focused and engaged in the present moment and in experience that we are living, without turning our thoughts to the past or future; sensory-perceptual sharpening, which consists in focusing on the sensory aspects that are stimulated in a certain experience (e.g., smells or sounds); behavioral expression, which covers all those physical expressions of happiness (laughing, jumping, clapping) that contribute to generating a positive cycle of pleasure; temporal awareness, for example, reminding ourselves that the experience will not last forever may spur us to enjoy it even more; counting blessings, i.e., experiencing gratitude for having had the opportunity to experience something positive; avoidance of kill-joy thinking, which refers to not focusing exclusively on negative aspects when we go through a difficult time. Use of savoring strategies is positively associated with variables related to individual wellbeing (e.g., self-esteem, life satisfaction, self-reported optimism), whereas lack of savoring skills is linked to depressive feelings, hopelessness, and anhedonia (Bryant, 2003; Joormann and Stanton, 2016).

### Interpersonal Emotion Regulation

Humans often seek to moderate their emotional states through the comfort and support of other individuals, thus engaging in a process referred to as interpersonal emotional regulation (Zaki and Williams, 2013). When people listen to and support someone’s feelings by showing empathy, solidarity, and sharing, they are actually helping them to regulate the intensity of the emotions they experience. Zaki and Williams (2013) identify two different processes that can support interpersonal emotional regulation: response-independent and response-dependent. The latter is based on the quality of feedback received from the person with whom the emotion is shared; in other words, when people express their sadness or joy to another person, they are only satisfied if that person responds favorably to the request to share. If the interlocutor shows indifference, then emotional regulation does not succeed. Response-independent processes, meanwhile, do not imply that the person with whom emotional states are shared must respond in a particular way: the fact of having talked with someone about one’s emotions, giving them a label and identifying their source, is enough in itself to allow people to feel regulated. As is the case in self-regulation, different strategies can be used in interpersonal regulation, depending on the goal one wants to achieve for a given emotional state. We refer to down-regulation processes when people respond in a way that attenuates and minimizes the intensity of the emotional experience shared by the communicative partner, while also weakening behavioral responses (Krompinger et al., 2008). In an experimental study, Pauw et al. (2019) asked participants to watch a video of a man crying over his partner’s cheating and asked them to record a supportive video message for him. Analysis revealed that when the situation required immediate emotional down-regulation (such as having to be ready to face an upcoming job interview), participants attempted to regulate the other through disengagement from the emotional experience by implementing distraction and suppression strategies. For example, by saying “Try not to think about her for now,” or “Don’t let your emotions take over.”

When, on the other hand, a person shares positive events with significant others (relatives, friends, partners) and they respond in a way that maximizes and prolongs the positive feelings and benefits experienced, we refer to the phenomenon of capitalization (Gable et al., 2004; Gable and Reis, 2010; Reis et al., 2010). Gable et al. (2004) found that communicating positive situations with significant others is associated with increased wellbeing and daily positive affect. The authors also showed that if significant others respond actively and constructively to capitalization attempts, benefits increase: in couples, this translates into greater wellbeing in some important aspects of the relationship, such as daily marital satisfaction and intimacy.

### Crying in Literature

Crying is one of the most common and universally recognized forms of expression through which people manifest and share their emotions. Crying results from the interaction between psychobiological, cognitive, and social processes (cf. Vingerhoets et al., 2000) and serves several functions. It has been suggested in the literature that crying may lead to a decrease in tension (cf. Vingerhoets et al., 2009) and promote the recovery of psychological balance (Rottenberg et al., 2003; Hendriks et al., 2007), although the empirical evidence supporting these hypotheses is conflicting. Some authors have investigated the interpersonal effects of this emotional expression. For example, Hendriks et al. (2008) conducted a survey with the purpose of studying how individuals respond when faced with a crying person, and had participants read six short vignettes depicting social situations. Specifically, three vignettes depicted unpleasant situations (e.g., talking to someone at a funeral or causing a car accident), while the other three depicted pleasant situations (e.g., meeting someone who had just become a parent). In each vignette there was a main character for participants to identify with, and another person who may or may not be crying. In most cases, participants reported that if they were faced with a crying interlocutor, they would exhibit more emotional support and fewer negative feelings toward him or her. Crying could therefore serve as a means of attracting attention, conveying requests for help and support, stimulating others to provide emotional support and thereby facilitating social bonding (Frijda, 1997; Kottler and Montgomery, 2001).

### Cultural Differences in Emotional Expression

Several studies (cf. Matsumoto, 1989, 1990; Safdar et al., 2009) have investigated cultural differences that may influence the display of emotions. In this regard, Ekman and Friesen (1969); Ekman (1972) proposed the existence of social rules, referred to as display rules, which govern the manifestation of emotions within different cultural groups, and which are learned by individuals through the socialization process. These rules direct emotional expression based on whether it is more or less acceptable within a given culture (Matsumoto et al., 1999) and establish how, and in what context, a person should express their emotions. The studies by Ekman (1972) and Friesen (1972) provided the first, which has become a milestone in the history of psychology, provided the first evidence of the existence of the “display rules.” The authors highlighted how American and Japanese people have a different way of expressing their emotions depending on the context: if a stranger was present during the viewing of a movie with the participants, the Japanese (unlike Americans) tended not to express negative emotions, disguising them with smiles. The presence of a stranger activated a set of unwritten, culturally learned rules in Japanese participants that regulated the expression of negative emotions. Ekman and Friesen’s study was later resumed by Matsumoto (1992), who compared the responses of American and Japanese people in reference to a task of facial expression recognition, finding results in keeping with those of previous studies. In the same research, Matsumoto found that Japanese participants were less able to recognize negative emotions than American subjects, while he found no significant differences in the recognition of positive emotions. To account for these differences, the author investigated cultural differences between the United States and Japan, focusing specifically on the concepts of individualism and collectivism. In cultures with an individualistic features (like most Western cultures), the primary values are those of individual autonomy, self-interest and success; it can be hypothesized that this is why levels of conformity to the group are lower and independence is greater instead: emotions can be experienced primarily as personal experiences, the expression of which is an individual’s right. In these cultures, for example, the manifestation of anger is tolerated and considered functional for the individual, as long as it is expressed in socially appropriate ways (Eid and Diener, 2001). Collectivistic cultures, on the other hand, would foster a greater level of conformity within the group, and collective needs would tend to take precedence over individual needs. In general, these cultures seem to urge the individual to maintain cohesion and harmony in the group, rather than to foster individual affirmation (Matsumoto, 1991; Noon and Lewis, 1992); where emotions are experienced as interactive experiences, reflecting the social context, their expression needs to be controlled. In particular, negative emotions may threaten group cohesion and are therefore discouraged. The expression of emotions such as anger or contempt would not be considered acceptable, as they threaten authority and harmony in the group (Miyake and Yamazaki, 1995). Regarding positive emotions, in individualistic cultures there would be a great attention to the pursuit of happiness and its individual expression: not expressing positive emotions could be considered a failure, and unhappiness would tend to have a strongly negative connotation. Conversely, in collectivist cultures, display rules might also filter out the expression of positive emotions, as expression is sometimes considered undesirable (see Eid and Diener, 2001).

A study by Safdar et al. (2009) examined the display rules of seven basic emotions in three different populations: American, Canadian, and Japanese. The goal of the research was to compare the display rules of emotional expression, both across and within cultures. The authors hypothesized that Canadians and Americans showed more approval toward the expression of both negative emotions such as anger and disgust, as well as positive emotions such as happiness and surprise, than Japanese participants, and that all three populations did not show particular differences regarding the expression of emotions such as sadness or fear. In keeping with the first hypothesis, the results showed that Japanese subjects expressed fewer negative emotions than Americans and Canadians, and showed significantly lower mean scores in the expression of positive emotions than Canadians. It was also found that there was no difference between Japanese and North Americans in the expression of emotions of sadness or fear.

Regarding the emotional expression of crying, some authors have conducted cross-cultural studies (see Kraemer and Hastrup, 1986; Vingerhoets and Becht, 1997). Williams and Morris (1996) investigated the crying behaviors of 448 participants from Great Britain and Israel, and the results showed that Britons cried more frequently than Israelis and that women cried more intensely and more often than men. Van Hemert et al. (2011) conducted a study to examine the tendency to cry in 37 countries, including Africa, Asia, the Caribbean, Europe, the Middle East, North America, Oceania, and South America. Analyses showed that crying is not significantly related to perceived distress, but is related to subjective wellbeing; that is, happier countries report more expressions of crying. At for the frequency, the results also showed that crying occurs more frequently in democratic countries, with an individualistic culture, where there is greater wealth and political freedom.

### Tears of Joy

TOJ is a special form of crying which can occur when a person is completely involved in situations in which they experience great joy. TOJ is an emotional experience that has been little researched to date, so much so that it is not even mentioned in an atlas of 156 human emotions (Watt Smith, 2015), and in the study conducted by Cowen and Keltner (2017) at the University of California at Berkeley—which identified 27 different interconnected emotions—it is not explicitly mentioned; it is shown in a video (this study made use of video recordings of different emotional expressions) within the category of the emotion of joy. The main studies in the literature that have focused on TOJ consider it as a dimorphous expression of positive emotions (cf. Aragón et al., 2015; Aragón, 2017; Aragón and Clark, 2017): the strong emotion felt (joy) is manifested through an expressive mode (crying) that refers to a different, or even opposite, emotional state (sadness, pain). An example of this is the tears shed by Olympic athletes after winning a gold medal: their joy is so enormous and uncontrollable that it triggers a crying reaction. The hypothesis put forward by the authors is that dimorphous expressions perform a regulatory function of emotional states: when individuals feel overwhelmed by positive feelings—so intense that they are perceived as unmanageable—they may respond through the expression of an emotion that has opposite value to the one experienced in order to compensate for its excess. In this regard, Aragón and Clark (2017) hypothesize that TOJ also function as a model of interpersonal emotional regulation. Through six experimental studies, the authors found that, when faced with joy expressed in a dimorphic way by significant others, people tend to provide down-regulation responses, thus dampening the intensity of the emotion and attempting to bring it to a level perceived as more manageable. Aragón (2017) again argues that dimorphous expressions cannot be identified with mixed expressions of emotion: as shown in their study, dimorphic expressions derive from a single evaluation of the event experienced, for which there is a single corresponding emotional experience, yet manifested through two recognized expressions, one with positive valence (smiling) and one with negative valence (crying). In contrast, mixed emotion expressions arise when an event stimulates positive and negative evaluations and emotions simultaneously (e.g., joy and sadness), which might then manifest in two emotional expressions of opposite meaning (Larsen et al., 2001; Larsen and McGraw, 2014). For example, Aragón in their study (ibid.) shows participants a video of of a generous man willing to help others; the video also informs viewers that the man died young in a tragic accident. From the analyses performed, the researcher finds that those who watched this video reported paired positive and negative appraisals, emotions, and expressions (joy/sadness, smiling/crying), in contrast with participants who watched the same video but were informed that the man had lived a long time; these latter participants reported primarily positive appraisals and positive emotions. Aragón and Bargh (2018) also suggest that dimorphous expressions may represent certain motivational orientations: in particular, happiness expressed through expressions of anger may communicate experiences of appetitive nature, characterized by the drive toward a desired end state; happiness manifested through expressions of sadness (such as tears) may communicate consummatory states, characterized by the hedonic experience of pleasure when the desired state has been achieved. For example, a basketball player may manifest happiness through aggressive expressions while he is playing a game and scores a point (appetitive state); the same athlete may manifest joy through tears when the desired state has been achieved, or when the game has been won (consummatory state).

An initial taxonomy of experiences in which TOJ occurs was developed by Hoffman et al. (2013). Through a systematic study using a sample of Indian adults, the researchers came to identify 15 main types of situations in which TOJ may occur: non-romantic affection; personal achievements; birth of a child; reunion; romantic love; identification with a movie/other medium; observing a child; reflection on one’s life; recovery of a loved one from illness/injury; personal recovery from illness/injury; aesthetic delight; material gain; individual religious experience; interpersonal laughter; witnessing an act of goodness. The research also found that the most frequently reported categories were those related to non-romantic affection and personal success, while religious experiences were reported less frequently. Subsequently, a more nuanced study conducted by Zickfeld et al. (2020) distinguished between four different macro-categories of positive tears: tears of affection (e.g., those shed during a wedding), tears of success (e.g., those shed for a sporting victory), tears of beauty (when one perceives something as extraordinarily beautiful), and tears of amusement (shed in situations in which one laughs so hard that one begins to cry). A number of international studies have investigated whether tears provoked by negative emotions show a different impact on mood compared to those stimulated by positive feelings, and have also tried to identify the possible functions of positive emotional crying. According to Ishii and Shinya (2021), positive crying is more effective in calming one’s mood than crying triggered by negative feelings, while Hoffman et al. (2016) find that the frequency of joyful tears is significantly associated with reduced self-perceived transitory stress, physical wellbeing following joyful crying, and self-reported health perceptions.

### The Meaning of Life in Literature

We conclude the literature review section by addressing the topic of MOL; the authors’ clinical experience has shown that patients tend to associate TOJ with the meaning of their lives. The literature (cf. King et al., 2006; Datu, 2015) shows that having a clear idea of the meaning of one’s own life is linked to people’s propensity to experience greater wellbeing and positive affect. In this regard, King et al. (2006) believe that it is the daily frequency of positive emotional states that may give meaning to a day or not. Other authors (Emmons, 2003; McDonald et al., 2012; Machell et al., 2015) relate MOL to the achievement of one’s own goals: individuals attach great value to these activities, and their accomplishment gives meaning to one’s actions, thus also increasing the sense of self-efficacy and promoting the development and maintenance of wellbeing (cf. Diener, 1984; Emmons, 1986). Machell et al. (2015) investigated how people’s everyday events are able to influence their perception of meaning on a daily basis. The researchers asked 162 college students to complete daily reports in which they provided measures of: meaning in life, positive and negative social events and achievements, and positive and negative affects. The results showed that social events and positive achievements were correlated with higher perceptions of meaning, whereas social events and negative achievements were correlated with lower perceptions of meaning. Existence may also be infused with meaning through social relationships and contact with others. In this regard, Lambert et al. (2013) investigated which element of social relationships was most likely to promote a sense of MOL. In one study, which included American and Indian participants (the latter in smaller numbers), four methodologically different studies were conducted that found that a strong sense of belonging correlated with higher levels of perceiving life as meaningful, much more so than social support and social worth.

### Methodological Background: Narratives and Narrative Psychology

Before describing the methodological approach of the research, we consider it useful to make a brief reference to the use of narratives, narrative psychology, and the biographical approach to the study of emotions and actions related to emotional manifestations. In recent years, the use of narratives in social research has become a popular and widely used approach in the humanities. Various models of narrative analysis have been developed from materials and research data based on biographies, biographical and narrative interviews, diaries, and—more recently—blogs and other web sources. Broadly speaking, narrative analyses are based on the philosophical assumptions of ontological relativism and constructionism (Smith, 2013); these models imply a continuous interaction between the researcher and the external environment and view knowledge (including scientific knowledge) as a co-constructed and culturally-characterized product. Narratives are methods rooted in people and contexts, situations and cultures; in this sense, using narratives has theoretical implications, both regarding theory of method (how to use narratives) and theoretical elaboration resulting from the analysis of results (what to do with narratives). The reference to narrative constructionism here recalls a socio-culturally oriented approach that understands human beings as “meaning makers” who make use of narratives to interpret, mediate, and share their experience in story form (Smith and Monforte, 2020); in this sense, a narrative is a story told by tracing cultural canons. The stories each of us tell about our experiences (including those elaborated from biographical experience) are based on a narrative structure (Sarbin, 1986a; Murray, 1995). The relationship between biography and self-narration is so close that real streams of study (such as narrative psychology) and research tools (such as biographical interviews) have become widespread in the history of psychology and psychological research. Mancuso and Sarbin (1983) argue that human beings think, perceive, imagine, and dream according to a narrative structure; each individual gives events an order and confers on them a plot which together lay the foundation for a narrative description of reality. Bruner (1990, 2002) states that every narrative can be considered a mental model, a way of perceiving and organizing reality; in order to understand human behavior, it is necessary to bring out the individual’s ability to narrate. Our experience takes a narrative form because actions and thought (cognitive processes such as, for example, planning and memory) are guided by narrative structures: the narrative, in short, is our way of organizing social episodes, actions and their stories, as is widely attested by autobiographical studies (Sarbin, 1986b; McAdams et al., 1996).

Given these premises, in the present article we decided to use a qualitative and narrative approach to explore, through the interviews, the psychological and cultural factors that influence the emotional representation of joy in a particular circumstance, that is, when it is expressed through unstoppable crying: TOJ. The guidelines for analysis of the interviews were shared among the three authors of this article; two coded the texts and extrapolated the results; one acted as a supervisor. The entire work was guided by the qualitative research validity criteria described by Tracy (2010), namely: (a) worthy topic (the topic of the research is relevant, timely, significant and interesting), (b) rich rigor (the study relies on sufficient, abundant, appropriate, and complex theoretical constructs, context issues and data collection and analysis processes), (c) sincerity (the study is characterized by a clear positioning of the researcher(s) in the field, transparency about the methods and challenges), (d) credibility (the research is marked by a thick description, concrete detail, and multivocality, (e) resonance (the research influences or moves particular readers or a variety of audiences through evocative representation of processes under investigation and shows transferable findings), (f) significant contribution (the research provides a significant contribution to the field), (g) ethics (the research considers situational and culturally specific ethical factors), and (h) meaningful coherence (the study achieves what it purports to be about, uses methods and procedures that fit its stated goals and meaningfully interconnects literature, research questions and findings, interpretations). The process of analysis, which leads to the elaboration of results from a qualitative study, is cyclical and iterative: the work of the researcher resembles a moving back and forth between each interview, between pieces of the same interview, giving greater emphasis to some linguistic forms and content today and to others tomorrow, once again under the banner of the complexity of qualitative work (Smith and Monforte, 2020).

## Research: Is There a Connection Between Tears of Joy and the Meaning of Life?

In the present research we wondered whether TOJ could be considered not merely an emotional expression, and therefore a sort of subcategory of joy, but a phenomenon with its own specific qualities; whether it was not merely an automatic emotional self-regulation but also fulfilled a specific adaptive function. The research hypothesis is that TOJ is an emotion that is not entirely superimposable with joy but that it accompanies and signals the most significant experiences in people’s lives, whereby the meaning of their lives is understood; its attainment constitutes the most ambitious goal that a human being can establish for themself in the orientation of their entire existence. To investigate this hypothesis we chose to use a qualitative method, through semi-structured interviews, to facilitate a free narrative of emotional experience in the respondents and to bring out potentially new aspects not yet investigated in the literature.

### Participants and Interviews

Our qualitative approach was based on the collection of in-depth, semi-structured interviews with 24 participants, of which 17 were from India, 6 from Japan, and 1 from England. The recruitment of participants may seem atypical. In fact, the entire research begun with interpersonal and friendly contacts with two cultural mediators who put one of the authors in contact with some interviewees (the interviews took place during a 6-month trip to India and Japan by one of the authors). At a later stage, the recruitment of the other interviewees followed a snowball principle, a strategy now recognized in qualitative research (Heckathorn, 1997; Morgan, 2008). The recruitment has, therefore, followed a mix of snowball-like organization and casual encounters; both solutions are accepted in qualitative research and mixed methods. Specifically: 8 chance encounters (5 Indian, 2 Japanese, 1 English), 12 encounters organized by a professor of Periyar University (Salem, Tamil Nadu, India) with students from the Faculty of Psychology, and 4 encounters organized by a cultural mediator from Tokyo. Although not belonging to the two cultures Indian and Japanese, we decided to keep the interview with an English man within the group because of his interesting contribution to the topic of research. All interviews were audio-recorded and conducted in English. If the interviewee was unable to speak English, the presence of a language mediator was requested (5 interviews were conducted with the presence of a mediator). An informed consent form was signed by each interviewee. In order to respect ethical and privacy issues we decided to anonymize all participants, so that the parts of the interview reported in this article are attributed to interviews and not to specific individuals; moreover, male personal pronouns are used everywhere, regardless of the gender of the interviewees. Regarding the demographic characteristics of the respondents: 15 women (11 Indian and 4 Japanese) and 9 men (6 Indian, 2 Japanese, 1 English) participated. The average age of the respondents was 29.2. The mean age of the Indians was 23.2; that of the Japanese was 42.5. This difference is consistent with the different overall mean age of the two populations: in India 28.7; in Japan 48.6^1^.

Interviews were conducted following a semi-structured protocol. The interview guide can be found in Appendix. As can be read in the interview guide text, the questions about TOJ were placed at the beginning of the interview, while those about the MOL were placed at the end, in order to reduce the possibility the interviewee associated the two topics only because they were treated one after the other. The interview was conducted in a style open to dialogue and following the narratives of the interviewees, so that it was possible to better understand the specificity of the socio-cultural context in which they lived. The approach used made it possible to co-construct a shared narrative between interviewee and interviewer while adhering to the cultural and local context in which the emotional expression was taking place. The value of co-construction in the data collection phase described by Bruner (1990) constitutes the main theoretical-methodological reference for the research.

### Qualitative Data Analysis

The semi-structured interviews were subjected to a qualitative content analysis through a coding procedure for narrative themes (Ryan and Russell Bernard, 2003; Braun and Clarke, 2006). Prior to content analysis, each interview was transcribed, and the interviewees’ transcripts reviewed by a language facilitator. Next, in each of the transcribed interviews, an attempt was made to identify emergent themes. In the tradition of the qualitative approach, a theme is important because it captures something significant about the data as it relates to the research questions (Braun and Clarke, 2006). We used MAXQDA software to perform computer-supported qualitative content analysis (Kuckartz, 2013; Kuckartz and Rädiker, 2019) to filter the structure of our transcripts and then code them. In the first step, major themes were identified through reading the interviews. This led to the identification of the main content analysis categories (codes and code sets):

1. Tears of joy (TOJ);
2. Meaning of life (MOL);
3. Personal characteristics.

After identifying the parts of the interview pertaining to each of these three thematic areas, we proceeded to extend the analysis, extrapolating segments of text from the transcripts which had specific, recognizable contents relating to the main topics:

1. TOJ: frequency, trigger events, description, situations in which it happened, situations in which it didn’t occur, tensions before, family/friends/social conflict, inner conflict.
2. MOL: whether life has a meaning or not, trigger events, what is the MOL, related concepts, connection between TOJ and MOL.
3. Personal characteristics: self-description (hard or soft), ways of experiencing emotions (whether they can or can’t control their emotions), whether they speak about themself in the third person, personal/professional realization.

Each interview segment featuring the above-described topics was (re)coded so as to detect, with greater precision, the specific narrative theme around which the discourse of the interviewees was built; for each theme the corresponding segment was identified and defined (coded). More dense segments (articulate, complex, polysemic) were coded with more than one code.

## Results

A first reflection concerns the different responses to the project among Indian and Japanese participants. A certain ease in recruiting Indian interviewees was contrasted by a greater difficulty in recruiting people that were happy to be interviewed by a foreigner in the Japanese context. In Japan, on several occasions the initial openness to the project was followed by disinvestment; during the interviews—due also to the difficulty of communicating in English—even those most open to dialogue showed some resistance to talking about emotional issues. What we observed is consistent with established findings in the literature about Japanese people’s reticence in expressing their emotions with strangers (Nakane, 1970; Matsumoto, 1991). The different emotional investment in the interviews between Indian and Japanese participants can also be seen through the different richness of the codes attributed during the qualitative analysis: the interviews of the Indian participants have an average of 34.9 codes attributed to each interview (limited to the themes of TOJ and the MOL); in the case of the Japanese participants the average number of codes per interview is 13.5. Another interesting finding is that almost all Japanese interviewees, specifically all those from a predominantly mountainous prefecture bordering Tokyo, initially confused the interviewer’s description of TOJ with a different type of emotional experience that they claimed they had experienced many times: feeling sadness inside and showing joy externally (the exact opposite of what happens with TOJ). This cultural duty to repress one’s negative emotions in favor of preserving group harmony is a finding consistent with what has been described in cultural anthropology texts (cft. Nakane, 1970).

### Tears of Joy

The interviews indicate that the Indian participants have generally experienced TOJ a few times, while the Japanese participants report that it has happened to them only once. In general, however, TOJ is a known experience, but not one experienced it with particular frequency; this could be due to the young age of most of the interviewees (Table 1). It is also significant to note that the participants who said they had never cried for joy were also more likely to describe themselves in a “hard” and confident way (e.g., “I have a different set of priorities,” “I’m very good at deleting people,” “I have been able to shield my personal ambitions. I’m very ambitious”). The reported contexts in which TOJ have been experienced are: with relatives, during a birthday, at university/school, during a competition, receiving news, doing hard work/solving a problem, at the birth of a child, in a religious building, in nature, during a political election, during a wedding, helping someone, cooking, watching a video, alone. The main triggers recognized by respondents were: achieving a result, pride for others, birth, positive surprise, acceptance by the family, a child being given his/her name, spiritual wonder, wedding, solving a critical situation, feeling part of a group (Table 2). Consistent with findings in the literature (Hoffman et al., 2013), the achievement of a personal success (winning a school or a sport competition, gaining access to a university or a job) is a major trigger of TOJ. Again, the fact that there is no co-prevalence of TOJ experiences associated with family life moments may depend on the young age of many of the respondents. The terms used to describe the TOJ experience were: instantaneous, instinctive, unexpected, incomprehensible, significant, powerful, emotional, shocking, overwhelming, relevant, with a big impact; experience of greatness, of change, of ecstasy, of victory, of achievement; insight, deep understanding, sense of responsibility; feeling loved, happy, proud, alive, free, important, special, on the right track. The three terms that stand out the most are “happiness,” “unexpected,” and “achievement”: the triggering event was unexpected, just as the strength of the reaction upon the achievement of an important goal for their life and career was also unexpected. Consistent with this, the main emotional tensions that the interviewees experienced before the TOJ were related to not knowing if they would be able (or if their family members would be able) to achieve a certain goal (“When you decide to participate in something, especially a competition, you always want to win, you know, there is always this ambition, desire behind. But you also know that you doubt yourself”), but also a sense of constraint due to a commitment made to oneself or one’s family (“I was feeling so stuck”), which was followed by a sense of liberation (Table 3).

**TABLE 1:** Frequencies of coded category “Tears of joy”.

TOJ: Frequencies	
Sometimes	9
Once	6
Never	6
Many times	2
I don’t remember	1

**TABLE 2:** Frequencies of coded category “Triggering events”.

TOJ: triggering events	Frequencies
Achieving a result	12
Pride for others	4
Birth	3
Positive surprise	3
Acceptance by the family	2
To a child is given his/her name	2
Spiritual wonder	2
Wedding	1
Solving a critical situation	1
Feeling part of a group	1

**TABLE 3:** Frequencies of coded category “Tension before tears of joy”.

TOJ: tension before	Frequencies
I didn’t know if I/he/she would be able to	7
I had to, but I didn’t want to	7
I/he/she had worked so hard	5
I didn’t know if it would be possible	3
I suffered for family pressure	3
I waited it for a long time	2
I was unhappy	2
I felt uncomfortable with others	1

### Achievement of Goals as the Main Cause of Tears of Joy

In particular, a strong correspondence was found between TOJ at the achievement of an important goal and the tension of not knowing if one would be able to achieve it; it is also curious to note that the three full words that appeared together most frequently in the interview texts are “[I] don’t know.” We might advance the hypothesis that the unexpected happiness of TOJ becomes more easily accessible to those who strongly desire to achieve a goal but are not fully sure of their abilities. We also found a co-presence of codes between TOJ caused by the realization of an important goal and the presence of an internal conflict (“So I tried to get some job, but mentally I am not in a normal stage”), the description of oneself as able to control one’s emotions (“I told you that I don’t cry very easily. So if I do get good news in front of people, I am just happy”), and the description of the meaning of one’s own life as “helping/having a good impact on others” (“So it’s our duty to live the life to the fullest and have a good life if possible helping others. Being good with others”).

### Meaning of Life: Connection With Tears of Joy

“Does life have meaning? If yes, what is the meaning of your life? Do you think life has meaning in and of itself, or are we the ones who put it in there?” When the interview was coming to a close and this question emerged, it always caught the interviewee off guard. It looked like the topic was not as common for them, and the answers were often more succinct than when the topic of emotions was addressed. Similarly, when asked if they saw any connection between their experiences of TOJ and their definition of MOL, an expression of surprise returned: they had never thought about it before, but yes, they did see a connection; respondents, both Indian and Japanese, leaned toward yes: life has meaning (Table 4). The MOL was defined according to the following categories: helping, having a positive impact on others, loving, having good relationships, achieving important goals, doing one’s best, having ambition, following the flow, happiness, a sense of wholeness, being healthy, worshiping God, being creative, and having experiences. Similar to the experiences of TOJ most commonly found in the literature, the terms that occur most frequently for defining MOL are “help/having good impact,” “love/good relationship,” “achievement/ambition/doing the best you can”: having good connections with others, and achieving personal life goals. We cry for joy for reasons connected to what we believe to be the main meaning of our lives. When asked to elaborate further on what they meant by “meaning of life” respondents used expressions such as: doing something good, connecting with others, God, death, making a difference, having an influence, finding the reason behind, one’s dignity, deciding for one’s dreams, improving oneself, changing one’s perspective, positivity, doing with what one has, something non-materialistic, personal ambitions.

**TABLE 4:** Frequencies of coded category “Life has a meaning or not”.

MOL: Life has a meaning or not?	
Yes	20
Yes and no	2
I don’t know	2

As we have said, there is a connection between TOJ and the MOL for respondents. However, not all respondents answered this question; those who did mention it leaned toward yes (Table 5). So much so that, even in the analysis of the relationship between codes, there is a strong co-presence between those who said “Sometimes I have cried for joy” and those who said “Yes, life has a meaning” (Table 6).

**TABLE 5:** Frequencies of coded category “Connection between TOJ and MOL”.

Is there a connection between TOJ and MOL?	
Yes	12
I don’t know	1

**TABLE 6:** Shared segments between tears of joy and meaning of life.

		Life has a meaning or not					

		Yes	Yes and no	I don’t know	
Tears of joy: frequency	Sometimes	8	1		9
	Once	6			6
	Never	4		2	6
	Many times	2			2
	I don’t remember		1		1
		20	2	2	

## Discussion and Conclusion

Through this research, participants had the opportunity to talk about their emotions. Conducting interviews allowed them to name unusual emotional manifestations, and link them to specific events in their lives. The use of narrative methods highlighted some typicalities of the dimorphous emotional expression of TOJ. This is an experience that has been lived by almost all of the interviewees, though not with particular frequency. The Indian participants reported having experienced TOJ a few times, while the Japanese participants reported more frequently that it had happened to them only once; this could be due to the cultural tendency, typical of Japanese people, to favor a greater control of emotional expressions. In this sense, those who reported never having cried for joy also tended to describe themselves as inflexible. The typical profile that emerged during the interviews is of a human being who cries for joy a few times, and who does so—consistently with the findings in the literature—when achieving an important result in their life. In addition, this is often when achieving an important result which they were not sure they would be able to achieve. The emotional experience of TOJ is completely unexpected for the person experiencing it and the triggering event is unexpected (e.g., the person did not expect that they would win a competition). The strength of the joyful reaction experienced upon achieving an important life goal is unexpected too. It was interesting to note that respondents spoke of an emotional tension—experienced prior to the TOJ—mainly due to not knowing whether they (themselves or a family member) would be able to achieve a given goal. Thus, it seems that the unexpected happiness of TOJ is more easily accessible to those who strongly desire to achieve a goal but do not feel completely confident in their abilities. The typical profile that emerged during the interviews is also a person who believes that life has a meaning; this person also believes that there is a strong connection between the MOL and the episodes in which they experienced TOJ. Both Indian and Japanese participants argued that—as is consistent with findings in the literature—the meaning of life corresponds with, on the one hand, having good relationships with others, helping them and having a good impact on them, and on the other hand in doing one’s best and accomplishing important goals. The main reasons why people cry for joy seem to have a close connection to the themes that people report when describing the meaning of their lives.

Given the data collected from the research, we believe we have identified some elements to support the hypothesis that TOJ can be considered not only a dimorphous emotional expression of joy, but also as an emotion in its own right with specific characteristics; moreover, the literature goes in the direction of indicating an increasing number of human emotions that are interconnected and have multiple nuances. The possible adaptive function of TOJ could be to direct people toward a higher level of wellbeing by pointing them to the types of experiences that make their lives meaningful. We conclude by pointing out that the present research contains all the typical limitations of qualitative research, including a very small sample of respondents and a greater freedom for both the interviewer and the respondent to “let oneself go” during the dialogue; so much so that some topics of the research were not discussed with all respondents. We hope that in future more space will be given to the study of TOJ and dimorphous emotional expressions, and that the connection between TOJ and people’s definitions of the MOL will be further investigated using both qualitative and quantitative methods.

## Data Availability Statement

The raw data supporting the conclusions of this article will be made available by the authors, without undue reservation.

## Ethics Statement

Ethical review and approval was not required for the study on human participants in accordance with the local legislation and institutional requirements. Written informed consent to participate in this study was provided by the participants.

## Author Contributions

BP designed the research, conducted the interviews, contributed to the interviews’ coding, and wrote the research section and the conclusions. RG conducted the literature research, transcribed interviews, contributed to the interviews’ coding, and wrote the literature sections. ED identified the methodological approach, contributed to the data analysis and interpretations, and wrote the methodological section. All authors have read and approved the final manuscript.

## Conflict of Interest

The authors declare that the research was conducted in the absence of any commercial or financial relationships that could be construed as a potential conflict of interest.

## Publisher’s Note

All claims expressed in this article are solely those of the authors and do not necessarily represent those of their affiliated organizations, or those of the publisher, the editors and the reviewers. Any product that may be evaluated in this article, or claim that may be made by its manufacturer, is not guaranteed or endorsed by the publisher.

## Footnotes

1
www.cia.gov/the-world-factbook/

## Appendix

### Text of the Semi-Structured Interview

I am an Italian psychologist. I’m going around the world to collect interviews on some psychological topics that relate to life, relationships, and important choices. Can I give you an interview? It will be completely anonymous. I will record the interview, which will last about 45 min. At the end I will ask you to sign a privacy form in which I declare the total protection and secrecy of your personal information.

The purpose of this interview is twofold: on the one hand to collect some information for research that I am conducting at the international level, on the other hand to help the interviewee to reflect on important aspects of his/her life on which, if anything, he/she had never thought before.

The first part of the interview is dedicated to emotions, and to one emotion in particular: tears of joy. This is a particular emotion because, on the one hand, if you look at the person’s face, he or she seems to be crying with sadness, but inside, instead, they feel joy. Have you ever experienced this type of emotion? (If the person didn’t understand what the emotion was, some typical episodes in which people cry for joy were suggested, such as at the birth of a child, or during a wedding, or winning a competition. If the person still did not understand, two pictures of people crying for joy were shown: Fedez and Chiara Ferragni at the birth of Leone, and Javier Zanetti with the Inter jersey raising his hands to the sky). How many times have you experienced this emotion? Could you describe some episodes in which it has happened? What did you feel at that moment? The fact that you cried with joy in those very situations, what does that say about you?

Going in the opposite direction, I’d like to talk about a different kind of emotion: boredom. Do you find yourself bored? How many times a week or month does it happen to you? If it happens to you every day, what percentage of the time does boredom happen to you during a day? In what situations does it happen to you? Why do you find yourself in those situations? What does it say about you that you are bored in those situations? Do you have any strategies you put in place to try to manage boredom? Which ones work best?

As I mentioned at the beginning, I’m a psychologist, and also a psychotherapist; that’s why I’m so interested in emotions. Are you familiar with psychotherapy? Do you know what it is and how it works? What is the image that people in general have of psychotherapy? What problems do you think people bring to psychotherapy?

In psychotherapy, especially in the kind of psychotherapy I use—Brief Therapy—there is a lot of focus on the effective and ineffective strategies that people put in place. So I’d like to know about two moments in your life: a first instance where you were really proud of the way you handled the situation; and a second instance, on the other hand, where you were not very proud of the way you handled the situation. Would you like to tell me about these two episodes? What strategies did you implement in the first case? And in the second?

Now there are the last two themes, somewhat unrelated to the previous ones, on which I would like to know your point of view. The first theme is about the meaning of life. What is the meaning of your life? Have you ever asked yourself that before? In your opinion, does the meaning of life exist in itself or do we invent/choose it? Is there any connection between the episodes in which you cried for joy and the meaning of your life? Finally, the last theme. Do you remember a story, fairy tale, nursery rhyme, or song from your childhood that your parents, or caregivers, used to put you to sleep? Would you like to tell/sing it to me?

## References

[B1] Aragón O. R. (2017). “Tears of joy” and “tears and joy?” personal accounts of dimorphous and mixed expressions of emotion. *Motiv. Emot.* 41 370–392. 10.1007/s11031-017-9606-x

[B2] Aragón O. R. Bargh J. A. (2018). “So happy i could shout!” and “So happy i could cry!” Dimorphous expressions represent and communicate motivational aspects of positive emotions. *Cogn. Emot.* 32 286–302. 10.1080/02699931.2017.1301388 28415957

[B3] Aragón O. R. Clark M. S. (2017). Tears of joy” & “smiles of joy” prompt distinct patterns of interpersonal emotion regulation. *Cogn. Emot.* 32 1–27. 10.1080/02699931.2017.1360253 28797202

[B4] Aragón O. R. Clark M. S. Dyer R. L. Bargh J. A. (2015). Dimorphous expressions of positive emotion: displays of both care and aggression in response to cute stimuli. *Psychol. Sci.* 26 259–273. 10.1177/0956797614561044 25626441

[B5] Braun V. Clarke V. (2006). Using thematic analysis in psychology. *Qual. Res. Psychol.* 3 77–101. 10.1191/1478088706qp063oa 32100154

[B6] Bruner J. (1990). *Acts of Meaning.* Cambridge: Harvard University Press.

[B7] Bruner J. (2002). *Making Stories. Law, Literature and Life.* New York, NY: Farrar, Strass e Grioux.

[B8] Bryant F. (2003). Savoring Beliefs Inventory (SBI): a scale for measuring beliefs about savouring. *J. Mental Health.* 12 175–196. 10.1080/0963823031000103489

[B9] Bryant F. B. (1989). A four-factor model of perceived control: avoiding, coping, obtaining, and savoring. *J. Pers.* 57 773–797. 10.1111/j.1467-6494.1989.tb00494.x

[B10] Bryant F. B. Smart C. M. King S. P. (2005). Using the past to enhance the present: boosting happiness through positive reminiscence. *J. Happiness Stud.* 6 227–260. 10.1007/s10902-005-3889-4

[B11] Bryant F. B. Veroff J. (2007). *Savoring: A New Model of Positive Experience.* Mahwah, NJ: Lawrence Erlbaum Associates Publishers.

[B12] Cowen A. S. Keltner D. (2017). Self-report captures 27 distinct categories of emotion bridged by continuous gradients. *Proc. Natl. Acad. Sci. U.S.A.* 114 E7900–E7909. 10.1073/pnas.1702247114 28874542 PMC5617253

[B13] Datu J. A. D. (2015). The synergistic interplay between positive emotions and maximization enhances meaning in life: a study in a collectivist context. *Curr. Psychol.* 35 459–466. 10.1007/s12144-015-9314-1

[B14] Diener E. (1984). Subjective well-being. *Psych. Bull.* 95 542–575. 10.1037/0033-2909.95.3.542 6399758

[B15] Eid M. Diener E. (2001). Norms for experiencing emotions in different cultures: inter- and intranational differences. *J. Pers. Soc. Psychol.* 81 869–885. 10.1037/0022-3514.81.5.869 11708563

[B16] Ekman P. (1972). “Universal and cultural differences in facial expressions of emotion,” in *Nebraska Symposium of Motivation*, Vol. 19, ed. Cole J. (Lincoln, NE: University of Nebraska Press).

[B17] Ekman P. Friesen W. V. (1969). The repertoire of nonverbal behavior: categories, origins, usage, and coding. *Semiotica* 1 49–98. 10.1515/semi.1969.1.1.49

[B18] Emmons R. A. (1986). Personal strivings: an approach to personality and subjective well-being. *J. Pers. Soc. Psychol.* 51 1058–1068. 10.1037/0022-3514.51.5.1058

[B19] Emmons R. A. (2003). “Personal goals, life meaning, and virtue: wellsprings of a positive life,” in *Flourishing: Positive Psychology and the Life Well-Lived*, eds Keyes C. L. M. Haidt J. (Washington, DC: American Psychological Association), 105–128. 10.1037/10594-005

[B20] Fredrickson B. L. (1998). What good are positive emotions? *Rev. Gen. Psychol.* 2 300–319. 10.1037/1089-2680.2.3.300 21850154 PMC3156001

[B21] Friesen W. V. (1972). *Cultural Differences in Facial Expressions in a Social Situation: an Experimental Test of the Concept of Display Rules.* Ph.D. thesis. San Francisco, CA: University of California.

[B22] Frijda N. H. (1997). “On the functions of emotional expression,” in *The (non)Expression of Emotions in Health and Disease*, eds Vingerhoets A. J. J. M. Van Bussel F. J. Boelhouwer A. J. W. (Tilburg: Tilburg University Press), 1–14.

[B23] Gable S. L. Reis H. T. (2010). Good news! Capitalizing on positive events in an interpersonal context. *Adv. Exp. Soc. Psychol.* 42 195–257. 10.1016/S0065-2601(10)42004-3

[B24] Gable S. L. Reis H. T. Impett E. A. Asher E. R. (2004). What do you do when things go right? The intrapersonal and interpersonal benefits of sharing positive events. *J. Pers. Soc. Psychol.* 87 228–245. 10.1037/0022-3514.87.2.228 15301629

[B25] Gross J. J. (1998). The emerging field of emotion regulation: an integrative review. *Rev. Gen. Psychol.* 2 271–299. 10.1037/1089-2680.2.3.271

[B26] Gross J. J. (2001). Emotion regulation in adulthood: timing is everything. *Curr. Dir. Psychol. Sci.* 10 214–219. 10.1111/1467-8721.00152

[B27] Gross J. J. (ed.) (2007). *Handbook of Emotion Regulation.* New York, NY: The Guilford Press.

[B28] Gross J. J. John O. P. (2003). Individual differences in two emotion regulation processes: implications for affect, relationships, and well-being. *J. Pers. Soc. Psychol.* 85 348–362. 10.1037/0022-3514.85.2.348 12916575

[B29] Heckathorn D. D. (1997). Respondent-driven sampling: a new approach to the study of hidden populations. *Soc. Prob.* 44 174–199. 10.2307/3096941

[B30] Hendriks M. C. P. Croon M. A. Vingerhoets A. J. J. M. (2008). Social reactions to adult crying: the help-soliciting function of tears. *J. Soc. Psychol.* 148 22–41. 10.3200/SOCP.148.1.22-42 18476481

[B31] Hendriks M. C. P. Rottenberg J. Vingerhoets A. J. J. M. (2007). Can the distress-signal and arousal-reduction views of crying be reconciled? Evidence from the cardiovascular system. *Emotion* 7 458–463. 10.1037/1528-3542.7.2.458 17516822

[B32] Hoffman E. Garg N. R. Gonzalez-Mujica J. (2013). Tears of joy in India. *Ind. J. Pos. Psychol.* 4 212–217.

[B33] Hoffman E. Tran A. Compton W. C. Sasaki H. (2016). Tears of joy among Japanese young adults: implications for counselling. *Asia Pac. J. Counsel. Psychother.* 7 1–11. 10.1080/21507686.2016.1214157

[B34] Ishii Y. Shinya Y. (2021). Positive emotions have different impacts on mood and sympathetic changes in crying from negative emotions. *Motiv. Emot.* 45, 530–542. 10.1007/s11031-021-09887-1

[B35] Joormann J. Stanton C. H. (2016). Examining emotion regulation in depression: a review and future directions. *Behav. Res. Ther.* 86 35–49. 10.1016/j.brat.2016.07.007 27492851

[B36] King L. A. Hicks J. A. Krull J. L. Del Gaiso A. K. (2006). Positive affect and the experience of meaning in life. *J. Pers. Soc. Psychol.* 90 179–196. 10.1037/0022-3514.90.1.179 16448317

[B37] Kottler J. A. Montgomery M. J. (2001). “Theories of crying,” in *Adult Crying: A Biopsychosocial Approach*, eds Vingerhoets A. J. J. M. Cornelius R. R. (Hove: Brunner-Routledge), 1–17.

[B38] Kraemer D. L. Hastrup J. L. (1986). Crying in natural settings. *Behav. Res. Ther.* 24 371–373. 10.1016/0005-7967(86)90199-3 3729909

[B39] Krompinger J. W. Moser J. S. Simons R. F. (2008). Modulations of the electrophysiological response to pleasant stimuli by cognitive reappraisal. *Emotion* 8 132–137. 10.1037/1528-3542.8.1.132 18266524

[B40] Kuckartz U. (2013). *Qualitative Text Analysis: A Guide to Methods, Practice and Using Software.* London: Sage. 10.4135/9781446288719

[B41] Kuckartz U. Rädiker S. (2019). *Analyzing Qualitative Data with MAXQDA.* Berlin: Springer. 10.1007/978-3-030-15671-8

[B42] Lambert N. M. Stillman T. F. Hicks J. A. Kamble S. Baumeister R. F. Fincham F. D. (2013). To belong is to matter: sense of belonging enhances meaning in life. *Pers. Soc. Psychol. Bull.* 39 1418–1427. 10.1177/0146167213499186 23950557

[B43] Langston C. A. (1994). Capitalizing on and coping with daily-life events: expressive responses to positive events. *J. Pers. Soc. Psychol.* 67 1112–1125. 10.1037/0022-3514.67.6.1112

[B44] Larsen J. T. McGraw A. P. (2014). The case for mixed emotions. *Soc. Pers. Psychol. Compass* 8 263–274. 10.1111/spc3.12108

[B45] Larsen J. T. McGraw A. P. Cacioppo J. T. (2001). Can people feel happy and sad at the same time? *J. Pers. Soc. Psychol.* 81 684–696. 10.1037/0022-3514.81.4.684 11642354

[B46] Lazarus R. S. (1991). *Emotion and Adaptation.* Oxford: Oxford University Press.

[B47] Machell K. A. Kashdan T. B. Short J. L. Nezlek J. B. (2015). Relationships between meaning in life, social and achievement events, and positive and negative affect in daily life. *J. Pers.* 83 287–298. 10.1111/jopy.12103 24749860

[B48] Mancuso J. C. Sarbin T. R. (1983). “The self-narrative in the enactment of roles,” in *Studies in Social Identity*, eds Sarbin T. R. Scheibe K. E. (New York, NY: Praeger), 233–253.

[B49] Matarazzo O. Zammuner V. L. (2009). *La Regolazione Delle Emozioni.* Bologna: il Mulino.

[B50] Matsumoto D. (1989). Cultural influences on the perception of emotion. *J. Cross Cult. Psychol.* 20 92–105. 10.1177/0022022189201006

[B51] Matsumoto D. (1990). Cultural similarities and differences in display rules. *Motiv. Emot.* 14 195–214. 10.1007/BF00995569

[B52] Matsumoto D. (1991). Cultural influences on facial expressions of emotion. *South. Comm. J.* 56 128–137. 10.1080/10417949109372824

[B53] Matsumoto D. (1992). American-Japanese cultural differences in the recognition of universal facial expressions. *J. Cross Cult. Psychol.* 23 72–84. 10.1177/0022022192231005

[B54] Matsumoto D. Kasri F. Kooken K. (1999). American–Japanese cultural differences in judgements of expression intensity and subjective experience. *Cogn. Emot.* 13 201–218. 10.1080/026999399379339

[B55] McAdams D. P. Hoffman B. J. Mansfield E. D. Day R. (1996). Themes of agency and communion in significant autobiographical scenes. *J. Pers.* 64 339–377. 10.1111/j.1467-6494.1996.tb00514.x 16958706

[B56] McDonald M. J. Wong P. T. Gingras D. T. (2012). “Meaning-in- life measures and development of a brief version of the personal meaning profile,” in *The Human Quest for Meaning: Theories, Research, and Applications*, 2nd Edn, ed. Wong P. T. P. (New York, NY: Routledge), 357–382.

[B57] Miyake K. Yamazaki K. (1995). “Self-conscious emotions: the psychology of shame, guilt, embarrassment, and pride,” in *Self-Conscious Emotions: The Psychology of Shame, Guilt, Embarrassment, and Pride*, eds Tangney J. P. Fischer K. W. (New York, NY: Guilford Press), 488–504.

[B58] Morgan D. (2008). “Snowball sampling,” in *The SAGE Encyclopedia of Qualitative Research Methods*, ed. Given L. (Thousand Oaks, CA: SAGE Publications Inc), 816–817.

[B59] Murray K. D. (1995). “Narratology,” in *Rethinking Psychology*, eds Smith J. A. Harré R. Van Langenhove L. (London: Sage), 179–195.

[B60] Nakane C. (1970). *Japanese Society. A Practical Guide to Understanding the Japanese Mindset and Culture.* North Clarendon, VT: Tuttle Publishing.

[B61] Noon J. M. Lewis J. R. (1992). Therapeutic strategies and outcomes: perspectives from different cultures. *Br. J. Med. Psychol.* 65 107–117. 10.1111/j.2044-8341.1992.tb01691.x 1633116

[B62] Pauw L. S. Sauter D. A. van Kleef A. Fischer A. H. (2019). Stop crying! The impact of situational demands on interpersonal emotion regulation. *Cogn. Emot.* 33 1587–1598. 10.1080/02699931.2019.1585330 30810482

[B63] Plutchik R. (2001). The nature of emotions. *Am. Sci.* 89 344–350. 10.1511/2001.4.344

[B64] Reis H. T. Smith S. M. Carmichael C. L. Caprariello P. A. Tsai F. F. Rodrigues A. (2010). Are you happy for me? How sharing positive events with others provides personal and interpersonal benefits. *J. Pers. Soc. Psychol.* 99 311–329. 10.1037/a0018344 20658846

[B65] Rottenberg J. Wilhelm F. H. Gross J. J. Gotlib I. H. (2003). Vagal rebound during resolution of tearful crying among depressed and nondepressed individuals. *Psychophysiology* 40 1–6. 10.1111/1469-8986.00001 12751799

[B66] Ruini C. (2017). *Positive Psychology in the Clinical Domains, Research and Practice.* Bologna: Springer International Publishing. 10.1007/978-3-319-52112-1

[B67] Ryan G. V. Russell Bernard H. (2003). Techniques to identify themes. *Field Meth.* 15 85–109. 10.1177/1525822X02239569

[B68] Safdar S. Friedlmeier W. Matsumoto D. Yoo S. H. Kwantes C. T. Kakai H. (2009). Variations of emotional display rules within and across cultures: a comparison between Canada, USA, and Japan. *Can. J. Behav. Sci.* 41, 1–10. 10.1037/a0014387

[B69] Sarbin T. R. (1986a). “The narrative as root metaphor for psychology,” in *Narrative Psychology. The Storied Nature of Human Conduct*, ed. Sarbin T. R. (New York, NY: Praeger), 3–21.

[B70] Sarbin T. R. (1986b). *Narrative Psychology. The Storied Nature of Human Conduct.* New York, NY: Praeger.

[B71] Smith B. (2013). Disability, sport and men’s narratives of health: a qualitative study. *Health Psychol.* 32 110–119. 10.1037/a0029187 23244531

[B72] Smith B. Monforte J. (2020). Stories, new materialism and pluralism: understanding, practising and pushing the boundaries of narrative analysis. *Meth. Psychol.* 2:100016. 10.1016/j.metip.2020.100016

[B73] Tooby J. Cosmides L. (2008). “The evolutionary psychology of the emotions and their relationship to internal regulatory variables,” in *Handbook of Emotions*, eds Lewis M. Haviland-Jones J. M. Barrett L. F. (New York, NY: Guilford Press), 114–137.

[B74] Tracy S. J. (2010). Qualitative quality: eight “big-tent” criteria for excellent qualitative research. *Qual. Inq.* 16 837–851. 10.1177/1077800410383121

[B75] Van Hemert D. A. van de Vijver F. J. R. Vingerhoets A. J. J. M. (2011). Culture and crying: prevalences and gender differences. *Cross Cult. Res. J. Comp. Soc. Sci.* 45 399–431. 10.1177/1069397111404519

[B76] Vingerhoets A. J. J. M. Becht M. C. (1997). “International study on adult crying: some first results,” in *Poster at the Annual Meeting of the American Psychosomatic Society*, Santa Fe, NM.

[B77] Vingerhoets A. J. J. M. Bylsma L. Rottenberg J. (2009). “Crying: a biopsychosocial phenomenon,” in *Tears in the Graeco-Roman World*, ed. Fogen T. (Berlin: de Guyter), 439–475.

[B78] Vingerhoets A. J. J. M. Cornelius R. R. Van Heck G. L. Becht M. C. (2000). Adult crying: a model and review of the literature. *Rev. Gen. Psychol.* 4 354–377. 10.1037/1089-2680.4.4.354

[B79] Watt Smith T. (2015). *The Book of Human Emotions. An Encyclopedia of Feeling From Anger to Wanderlust.* London: Wellcome Collection.

[B80] Williams D. G. Morris G. H. (1996). Crying, weeping or tearfulness in British and Israeli adults. *Br. J. Psychol.* 87 479–505. 10.1111/j.2044-8295.1996.tb02603.x 8794556

[B81] Zaki J. Williams W. C. (2013). Interpersonal emotion regulation. *Emotion* 13 803–810. 10.1037/a0033839 24098929

[B82] Zickfeld J. Seibt B. Lazarevic L. Zezelj I. Vingerhoets A. J. J. M. (2020). A model of positive tears. *PsyArXiv* [Preprint]. 10.31234/osf.io/sf7pe

